# Determinants of surgeons’ adherence to preventive intraoperative measures of surgical site infection in Gaza Strip hospitals: a multi-centre cross-sectional study

**DOI:** 10.1186/s12893-020-0684-4

**Published:** 2020-01-30

**Authors:** Mohamedraed Elshami, Bettina Bottcher, Issam Awadallah, Ahmed Alnaji, Basel Aljedaili, Haytham Abu Sulttan, Mohamed Hwaihi

**Affiliations:** 1 Physician, Ministry of Health, Gaza, Palestine; 2 Graduate Student, Harvard Medical School, Boston, MA USA; 3 Faculty of Medicine, Islamic University of Gaza, Gaza, Palestine; 4 Shifaa Hospital, Gaza, Palestine; 5 Nasser Hospital, Gaza, Palestine; 6 European Gaza Hospital, Gaza, Palestine

**Keywords:** Surgical site infection, Wound infection, Gaza, Palestine, Infection control, Patient safety, Adherence, Guidelines

## Abstract

**Background:**

Surgical site infection (SSI) is one of the most common hospital-acquired infections and is associated with serious impact on the rates of morbidity, mortality as well as healthcare costs. This study examined factors influencing the application of several intraoperative preventive measures of SSI by surgeons and surgical residents in the Gaza Strip.

**Methods:**

A cross-sectional study was conducted from December 2016 to February 2017 at the operation rooms of the three major hospitals located in the Gaza-Strip, Palestine. Inclusion criteria for patients were being adult (aged ≥18 years), no history of wound infection at time of operation and surgical procedure under general anaesthesia with endotracheal intubation. The association between different patient- and procedure-related SSI risk factors and adherence to several intraoperative SSI preventive measures was tested.

**Results:**

In total, 281 operations were observed. The mean patient age ± standard deviation (SD) was 38.4 ± 14.6 years and the mean duration of surgery ± SD was 58.2 ± 32.1 minutes. A hundred-thirty-two patients (47.0%) were male. Location and time of the operation were found to have significant associations with adherence to all SSI preventive measures except for antibiotic prophylaxis. Type of operation had a significant association with performing all measures except changing surgical instruments. Patient age did not have a statistically significant association with adherence to any measure.

**Conclusion:**

The results suggest that the surgeon could be a major factor that can lead to a better outcome of surgical procedures by reducing postoperative complications of SSI. Operating department professionals would benefit from clinical guidance and continuous training, highlighting the importance of persistent implementation of SSI preventive measures in everyday practice to improve the quality of care provided to surgical patients.

## Background

Surgical site infection (SSI) is defined as “*an infection related to an operative procedure that occurs at or near the surgical incision within 30 days”* [1].

It is one of the most common hospital-acquired infections and is associated with serious impacts on the rates of morbidity, mortality and healthcare costs [2].

An international study reported higher SSI rates in low- and middle-income countries (LMICs) compared to high-income countries (23.2 and 14.0% vs. 9.4%), even after adjustment for factors related to patients, procedures and hospitals [3]. Interestingly, a previous study showed that despite the poor economic circumstances in Palestine, the SSI rate was comparable to that of high-income countries (9.6% vs 9.4%) [4].

There are many risk factors known to predispose to SSI. Some of them are related to the patient, like gender, age and high American Society of Anaesthesiologists (ASA) score, which is a score categorizing patients in six categories from I (normal healthy patient) to category VI (braindead patient, whose organs maybe harvested) [5]. Other factors were found to be about the characteristics of the operation itself such as long duration, high intraoperative contamination and emergency procedures [6].

The serious consequences experienced by patients who developed SSI determine the need for efforts to create strategies for the prevention of this infection, hence the importance of determining these SSI risk factors to minimize postoperative complications [7]. Furthermore, several simple but critical intraoperative preventive measures can lower the incidence of SSI [8]. The World Health Organization (WHO) developed some evidence-based recommendations for this purpose, taking into account the balance between benefit and harm, the quality of evidence level, cost-effectiveness, availability of resources, and patient values and preferences [8]. However, despite the inclusion of strongly graded recommendations in their guidelines, none of these could be based on high-quality evidence, which is lacking in support of most interventions. In fact, none of the existing evidence is derived from LMICs, leading to uncertainty about their effectiveness in implementing measures preventing SSI [9]. Many potential barriers to implementing the WHO guidelines in LMICs remain, including in the Gaza Strip, Palestine. Severe shortages of resources and facilities are a constant challenge such as inadequate environmental hygienic conditions; poor infrastructure; insufficient equipment; understaffing; paucity of knowledge and application of basic infection-control measures; prolonged and inappropriate use of invasive devices; overuse of antibiotics; and lack of local guidelines and policies [10, 11].

The surgeons’ recognition of the SSI risk factors may have an effect on their adherence to the SSI preventive measures. Although the importance of these measures is well described in the literature, few studies discussed the routine implementation of these measures during surgical procedures [12] and to the best knowledge of the authors, so far no study has tested their effectiveness. This study examined factors influencing the application of several intraoperative SSI preventive measures by surgeons and surgical residents at three major hospitals in the Gaza Strip, Palestine. Furthermore, it aimed to identify potential obstacles and possible improvements for achieving a better quality and safety of surgical procedures.

## Methods

### Study design

This was a cross-sectional descriptive study conducted from December 2016 to February 2017 at the operation rooms of the three major hospitals located in the Gaza Strip, Palestine. For the purpose of anonymising the results of this study, each of the three hospitals was given a symbol of either X, Y or Z. This will also support the focus on improving the quality of care and patient safety instead of only targeting specific hospitals.

### Study population and sample

Annually, around 23,000 surgical operations are performed in the study hospitals [13]. Patient inclusion criteria were: being an adult (aged ≥18 years), having no history of wound infection at the time of operation and having a surgical procedure under general anaesthesia with endotracheal intubation. Based on these criteria, convenience sampling was used to recruit 281 operations from the 5755 eligible operations (4.9%) during the study period at the three hospitals.

Based on the WHO 2016 recommendations, a data collection sheet was created and reviewed by several surgeons to test its comprehensiveness. Data were collected by direct observation of surgeons and surgical residents at the time of operation. Both, the observed staff and the data collectors, were blinded to the true research question (are patient- and procedure-related factors associated with the application of intra-operative SSI preventive measures by surgical staff?). As shown in previous studies, keeping the staff unaware of observations is intended to limit the “Hawthorne Effect”, when clinicians adjust their practice because they know that they are being observed [14, 15]. The data collectors were also blinded to minimize the ascertainment bias that can happen because of their knowledge about SSI risk factors. Therefore, the data collected were observations by data collectors, who recorded what they observed, rather than judgements of appropriateness at the time of data collection.

The data collected included: demographic characteristics (age, gender), operation details (type, duration, ASA score, urgency, intraoperative contamination), preventive measures of SSI before surgical incision [use of surgical antibiotic prophylaxis within 120 min pre-incision, at the time of incision or at both times, hair removal by shaving, surgical site skin preparation, surgical hand preparation, maintaining normal body temperature (normothermia)] and before skin closure [giving 80% fraction of inspired oxygen (FiO_2_), using povidone-iodine for irrigation of the incisional wound, double-gloving or change of gloves, and change of instruments for fascial, subcutaneous and skin closure].

### Statistical analysis

Statistical analyses were performed using the Statistical Package for the Social Sciences (SPSS) version 22 (IBM Corp, Chicago, Illinois). Descriptive statistics including frequencies and percentages for demographic data and the performed preventive measures were calculated. Age was categorized into three groups based on the relative risk of each one with the incidence of SSI described by previous studies [16, 17]. The same concept was applied to categorizing the duration of surgical procedures [18, 19].

Chi-square test was used to test the association between present SSI risk factors and performing the SSI preventive measures before making the surgical incision and before closure of skin with a *p*-value of ≤0.05 as a reference for statistically significant associations.

### Ethical considerations

No institutional review boards exist in the Gaza Strip. Ethical approval for this study was obtained from the Human Resources Department of the Palestinian Ministry of Health, which is the body in Gaza to issue ethical approvals for studies involving humans. Written informed consent was taken from patients before undergoing surgery. All data were collected and kept anonymously.

## Results

During the study period, a total of 281 operations were enrolled. The mean patient age ± standard deviation (SD) was 38.4 ± 14.6 years and 132 patients (47.0%) were male. The mean duration of surgery ± SD was 58.2 ± 32.1 min with only four operations (1.4%) lasting more than 2 h (Table 1)

**Table 1:** Summary of characteristics of study patients and surgical operations (*n* = 281)

Characteristic	n (%)
Gender	
Male	132 (47.0)
Female	149 (53.0)
Age	
18–25	73 (26.0)
26–64	196 (69.8)
65–80	12 (4.3)
ASA classification score	
I (normal healthy patient)	183 (65.1)
II (patient with mild systemic disease)	62 (22.1)
III (patient with severe systemic disease but not life-threatening)	25 (8.9)
IV (patient with severe systemic life-threatening disease)	11 (3.9)
Type of operation	
Abdominal exploration	12 (4.3)
Open appendectomy	46 (16.4)
Laparoscopic cholecystectomy	59 (21.0)
Anorectal surgeries	22 (7.8)
Open hernia repair	38 (13.5)
Thyroidectomy	22 (7.8)
Mastectomy	22 (7.8)
Lymph node excision	15 (5.3)
Vascular surgeries	45 (16.0)
Duration of operation	
≤ 30 min	67 (23.8)
31 to 60 min	130 (46.3)
61 to 90 min	39 (13.9)
91 to 120 min	41 (14.6)
> 120 min	4 (1.4)
Urgency	
Elective	208 (74.0)
Emergency	73 (26.0)
Intraoperative contamination	
Clean	144 (51.2)
Clean-contaminated	84 (29.9)
Contaminated	36 (12.9)
Dirty	17 (6.0)
Hospital	
X	111 (39.5)
Y	109 (38.8)
Z	61 (21.7)
Time of operation	
December 2016	83 (29.5)
January 2017	87 (31.0)
February 2017	111 (39.5)

*n* number of patients tested, *ASA* American Society of Anaesthesiologists

Of the included operations, surgical antibiotic prophylaxis was given to 271 patients (96.4%); of these, 125 (44.5%) received the antibiotics in the operating theatre just before incision, 116 (41.3%) 2 h before surgery and 30 (10.7%) at both times. Prior to 47 operations (16.7%), skin was prepared using aqueous povidone-iodine solution, whereas alcohol-based chlorhexidine gluconate solution was used in skin preparation of 234 operations (83.3%). Furthermore, hand preparation was performed before all operations with an antimicrobial soap and water used before 141 operations (50.2%) and a suitable alcohol-based handrub before the rest (*n* = 140, 49.8%). Maintaining normothermia by controlling room temperature using air conditioning was done in a quarter of operations (Table 2).

**Table 2:** Summary of frequency and percentages of the surgeons’ practice of the preventive intra-operative measures of SSI

Measure	n (%) (Total = 281)	WHO Recommendation	Strength^a^	Quality of Evidence^b^
Surgical antibiotic prophylaxis	271 (96.7)	The panel recommends the administration of SAP within 120 min before incision, while considering the half-life of the antibiotic.	Strong	Moderate
At time of incision	125 (44.5)			
Within 2 h pre-incision	116 (41.3)			
Both	30 (10.7)			
Hair removal by shaving	75 (26.7)	The panel recommends that in patients undergoing any surgical procedure, hair should either not be removed or, if absolutely necessary, it should be removed only with a clipper. Shaving is strongly discouraged at all times, whether preoperatively or in the OR.	Strong	Moderate
By the patient	72 (25.6)			
By the surgeon	3 (1.1)			
Skin preparation by alcohol-based antiseptic solutions based on CHG	234 (83.3)	The panel recommends alcohol-based antiseptic solutions based on CHG for surgical site skin preparation in patients undergoing surgical procedures.	Strong	Low to moderate
Hand preparation	281 (100.0)	The panel recommends that surgical hand preparation should be performed by scrubbing with either a suitable antimicrobial soap and water or usng a suitable alcohol-based handrub before donning sterile gloves.	Strong	Moderate
Maintaining normothermia	70 (24.9)	The panel suggests the use of warming devices in the OR and during the surgical procedure for patient body warming with the purpose of reducing SSI.	Conditional	Moderate
Giving 80% FiO_2_	156 (55.5)	The panel recommends that adult patients undergoing general anaesthesia with endotracheal intubation for surgical procedures should receive an 80% fraction of inspired oxygen intraoperatively.	Strong	Moderate
Irrigation of incision with povidone-iodine before closure	111 (39.5)	The panel suggests considering the use of irrigation of the incisional wound with an aqueous PVP-I solution before closure for the purpose of preventing SSI, particularly in clean and clean-contaminated wounds.	Conditional	Low
Gloving	106 (37.7)	The panel decided not to formulate a recommendation due to the lack of evidence to assess whether double-gloving or a change of gloves during the operation or the use of specific types of gloves are more effective in reducing the risk of SSI.	NA	NA
Double-gloving	91 (32.4)			
Change of gloves	9 (3.2)			
Both	6 (2.1)			
Change of surgical instruments	5 (1.8)	The panel decided not to formulate a recommendation on this topic due to the lack of evidence.	NA	NA

*n* number of patients tested, *WHO* World Health Organization, *SAP* surgical antibiotic prophylaxis, *OR* operating room, *CHG* chlorhexidine gluconate, *SSI* surgical site infection, *FiO*_*2*_ fraction of inspired oxygen, *PVP-I* povidone-iodine, *NA* not applicable

^a, b^based on the WHO guidelines

It was noted that the type of operation had a statistically significant association with adherence to all SSI intraoperative preventive measures performed before making the surgical incision (Table 3). In addition, performing operations in different hospitals and at different times was found to be significantly associated with adherence to these measures except for giving surgical antibiotic prophylaxis. However, no association was found between patient age and adherence to any preventive measures. Interestingly, the choice to use anti-microbial soap and water or alcohol-based handrub for hand preparation before surgery was significantly associated with the ASA score of the patient.

**Table 3:** Summary of associations between patients’ and operations’ characteristics with performing the SSI preventive measures before making the surgical incision (*n* = 281)

	Surgical antibiotic prophylaxis			Hair removal			Skin preparation by alcohol-based antiseptic solutions based on CHG			Hand preparation			Maintaining normothermia		
	Yes	No	p	Yes	No	p	Yes	No	p	By soap and water, n (%)	By alcohol-based rub, n (%)	p	Yes	No	p
	n (%)	n (%)		n (%)	n (%)		n (%)	n (%)					n (%)	n (%)	
Gender															
Male	125 (94.7)	7 (5.3)	0.20	66 (50.0)	66 (50.0)	**< 0.001**	111 (84.1)	21 (15.9)	0.75	67 (50.8)	65 (49.2)	0.91	25 (18.9)	107 (81.1)	**0.038**
Female	146 (98.0)	3 (2.0)		9 (6.0)	140 (94.0)		123 (82.6)	26 (17.4)		74 (49.7)	75 (50.3)		45 (30.2)	104 (69.8)	
Age-group															
18–25	70 (95.9)	3 (4.1)	0.78	19 (26.0)	54 (74.0)	0.98	62 (84.9)	11 (15.1)	0.91	32 (43.8)	41 (56.2)	0.123	25 (34.2)	48 (65.8)	0.10
26–64	189 (96.4)	7 (3.6)		53 (27.0)	143 (73.0)		162 (82.7)	34 (17.3)		100 (51.0)	96 (49.0)		42 (21.4)	154 (78.6)	
65–80	12 (100.0)	0		3 (25.0)	9 (75.0)		10 (83.3)	2 (16.7)		9 (75.0)	3 (25.0)		3 (25.0)	9 (75.0)	
ASA classification															
I	176 (96.2)	7 (3.8)		48 (26.2)	135 (73.8)		146 (79.8)	37 (20.2)		81 (44.3)	102 (55.7)		53 (29.0)	130 (71.0)	
II	59 (95.2)	3 (4.8)	0.65	22 (35.5)	40 (64.5)	0.07	53 (85.5)	9 (14.5)	0.07	30 (48.4)	32 (51.6)	**< 0.001**	14 (22.6)	48 (77.4)	0.07
III	25 (100.0)	0		5 (20.0)	20 (80.0)		24 (96.0)	1 (4.0)		20 (80.0)	5 (20.0)		2 (8.0)	23 (92.0)	
IV	11 (100.0)	0		0	11 (100.0)		11 (100.0)	0		10 (90.9)	1 (9.1)		1 (9.1)	10 (90.9)	
Type of operation															
Abdominal exploration	12 (100.0)	0		3 (25.0)	9 (75.0)		12 (100.0)	0		2 (16.7)	10 (83.3)		4 (33.3)	8 (66.7)	
Open appendectomy	46 (100.0)	0		21 (45.7)	25 (54.3)		41 (89.1)	5 (10.9)		14 (30.4)	32 (69.6)		11 (23.9)	35 (76.1)	
Lap. cholecystectomy	58 (98.3)	1 (1.7)		9 (15.3)	50 (84.7)		42 (71.2)	17 (28.8)		30 (50.8)	29 (49.2)		15 (25.4)	44 (74.6)	
Anorectal surgeries	21 (95.5)	1 (4.5)		4 (18.2)	18 (81.8)		16 (72.7)	6 (27.3)		12 (54.5)	10 (45.5)		8 (36.4)	14 (63.6)	
Open hernia repair	36 (94.7)	2 (5.3)	**< 0.001**	23 (60.5)	15 (39.5)	**< 0.001**	29 (76.3)	9 (23.7)	**0.005**	17 (44.7)	21 (55.3)	**< 0.001**	4 (10.5)	34 (89.5)	**0.002**
Thyroidectomy	21 (95.5)	1 (4.5)		0	22 (100.0)		18 (81.8)	4 (18.2)		10 (45.5)	12 (54.5)		8 (36.4)	14 (63.6)	
Mastectomy	22 (100.0)	0		0	22 (100.0)		18 (81.8)	4 (18.2)		6 (27.3)	16 (72.7)		10 (45.5)	12 (54.5)	
Lymph node excision	10 (66.7)	5 (33.3)		0	15 (100.0)		13 (86.7)	2 (13.3)		5 (33.3)	10 (66.7)		7 (46.7)	8 (53.3)	
Vascular surgeries	45 (100.0)	0		15 (33.3)	30 (66.7)		45 (100.0)	0		45 (100.0)	0		3 (6.7)	42 (93.3)	
Duration of operation															
≤ 30 min	62 (92.5)	5 (7.5)		11 (16.4)	56 (83.6)		61 (91.0)	6 (9.0)		30 (44.8)	37 (55.2)		25 (37.3)	42 (62.7)	
31 to 60 min	126 (96.9)	4 (3.1)	0.31	46 (35.4)	84 (64.6)	**0.007**	115 (88.5)	15 (11.5)	**0.001**	63 (48.5)	67 (51.5)	0.41	25 (19.2)	105 (80.8)	**0.042**
61 to 90 min	38 (97.4)	1 (2.6)		11 (28.2)	28 (71.8)		26 (66.7)	13 (33.3)		24 (61.5)	15 (38.5)		8 (20.5)	31 (79.5)	
91 to 120 min	41 (100.0)	0		5 (12.2)	36 (87.8)		29 (70.7)	12 (29.3)		21 (51.2)	20 (48.8)		12 (29.3)	29 (70.7)	
> 120 min	4 (100.0)	0		2 (50.0)	2 (50.0)		3 (75.0)	1 (25.0)		3 (75.0)	1 (25.0)		0	4 (100.0)	
Urgency															
Elective	199 (95.7)	9 (4.3)	0.46	49 (23.6)	159 (76.4)	0.06	166 (79.8)	42 (20.2)	**0.01**	114 (54.8)	94 (45.2)	**0.01**	53 (25.5)	155 (74.5)	0.76
Emergency	72 (98.6)	1 (1.4)		26 (35.6)	47 (64.4)		68 (93.2)	5 (6.8)		27 (37.0)	46 (63.0)		17 (23.3)	56 (76.7)	
Intraoperative contamination															
Clean	137 (95.1)	7 (4.9)	0.45	39 (27.1)	105 (72.9)	0.98	125 (86.8)	19 (13.2)	**0.008**	78 (54.2)	66 (45.8)	**0.002**	32 (22.2)	112 (77.8)	0.55
Clean-contaminated	82 (97.6)	2 (2.4)		23 (27.4)	61 (72.6)		61 (72.6)	23 (27.4)		43 (51.2)	41 (48.8)		21 (25.0)	63 (75.0)	
Contaminated	36 (100.0)	0		9 (25.0)	27 (75.0)		31 (86.1)	5 (13.9)		19 (52.8)	17 (47.2)		12 (33.3)	24 (66.7)	
Dirty	16 (94.1)	1 (5.9)		4 (23.5)	13 (76.5)		17 (100.0)	0		1 (5.9)	16 (94.1)		5 (29.4)	12 (70.6)	
Hospital															
X	110 (99.1)	1 (0.9)	0.12	27 (24.3)	84 (75.7)	**< 0.001**	65 (58.6)	46 (41.4)	**< 0.001**	105 (94.6)	6 (5.4)	**< 0.001**	4 (3.6)	107 (96.4)	**< 0.001**
Y	104 (95.4)	5 (4.6)		46 (42.2)	63 (57.8)		109 (100.0)	0		31 (28.4)	78 (71.6)		11 (10.1)	98 (89.9)	
Z	57 (93.4)	4 (6.6)		2 (3.3)	59 (96.7)		60 (98.4)	1 (1.6)		5 (8.2)	56 (91.8)		55 (90.2)	6(9.8)	
Time of operation															
December 2016	79 (95.2)	4 (4.8)	0.14	39 (47.0)	44 (53.0)	**< 0.001**	82 (98.8)	1 (1.2)	**< 0.001**	32 (38.6)	51 (61.4)	**0.001**	3 (3.6)	80 (96.4)	**< 0.001**
January 2017	82 (94.3)	5 (5.7)		24 (27.6)	63 (72.4)		75 (86.2)	12 (13.8)		58 (66.7)	29 (33.3)		16 (18.4)	71 (81.6)	
February 2017	110 (99.1)	1 (0.9)		12 (10.8)	99 (89.2)		77 (69.4)	34 (30.6)		51 (45.9)	60 (54.1)		51 (45.9)	60 (54.1)	

*n* number of patients tested, *CHG* chlorhexidine gluconate, *ASA* American Society of Anaesthesiologists

Note: *p*-values in bold are statistically significant

The adherence to preventive measures before skin closure, was found to be significantly associated with the location (hospital), time and urgency of the surgical procedure. Furthermore, the type of operation and its class of contamination were significantly associated with adherence to all SSI preventive measures, except for change of surgical instruments. On the other hand, age-group was not associated with adherence to any of these measures (Table 4).

**Table 4:** Summary of associations between patients’ and operations’ characteristics with performing the SSI preventive measures before skin closure (n = 281)

	Giving 80% FiO_2_			Irrigation of incision with povidone-iodine			Change of and/or double gloves			Change of instruments		
	Yes	No	p	Yes	No	p	Yes	No	p	Yes	No	p
	n (%)	n (%)		n (%)	n (%)		n (%)	n (%)		n (%)	n (%)	
Gender												
Male	58 (43.9)	74 (56.1)	**< 0.001**	60 (45.5)	72 (54.5)	0.07	61 (46.2)	71 (53.8)	**0.007**	4 (3.0)	128 (97.0)	0.19
Female	98 (65.8)	51 (34.2)		51 (34.2)	98 (65.8)		45 (30.2)	104 (69.8)		1 (0.7)	148 (99.3)	
Age-group												
18–25	39 (53.4)	34 (46.6)	0.83	35 (47.9)	38 (52.1)	0.14	26 (35.6)	47 (64.4)	0.31	3 (4.1)	70 (95.9)	0.21
26–64	111 (56.6)	85 (43.4)		70 (35.7)	126 (64.3)		73 (37.2)	123 (62.8)		2 (1.0)	194 (99.0)	
65–80	6 (50.0)	6 (50.0)		6 (50.0)	6 (50.0)		7 (58.3)	5 (41.7)		0	12 (100.0)	
ASA classification												
I	115 (62.8)	68 (37.2)		71 (38.8)	112 (61.2)		56 (30.6)	127 (69.4)		4 (2.2)	179 (97.8)	
II	35 (56.5)	27 (43.5)	**< 0.001**	25 (40.3)	37 (59.7)	0.75	29 (46.8)	33 (53.2)	**0.001**	1 (1.6)	61 (98.4)	0.84
III	4 (16.0)	21 (84.0)		9 (36.0)	16 (64.0)		17 (68.0)	8 (32.0)		0	25 (100.0)	
IV	2 (18.2)	9 (81.8)		6 (54.5)	5 (45.5)		4 (36.4)	7 (63.6)		0	11 (100.0)	
Type of operation												
Abdominal exploration	4 (33.3)	8 (66.7)		10 (83.3)	2 (16.7)		10 (83.3)	2 (16.7)		1 (8.3)	11 (91.7)	
Open appendectomy	14 (30.4)	32 (69.6)		32 (69.6)	14 (30.4)		23 (50.0)	23 (50.0)		3 (6.5)	43 (93.5)	
Lap. cholecystectomy	50 (84.7)	9 (15.3)		22 (37.3)	37 (62.7)		14 (23.7)	45 (76.3)		0	59 (100.0)	
Anorectal surgeries	20 (90.9)	2 (9.1)		6 (27.3)	16 (72.7)		5 (22.7)	17 (77.3)		0	22 (100.0)	
Open hernia repair	20 (52.6)	18 (47.4)	**< 0.001**	16 (42.1)	22 (57.9)	**< 0.001**	20 (52.6)	18 (47.4)	**0.001**	1 (2.6)	37 (97.4)	0.14
Thyroidectomy	20 (90.9)	2 (9.1)		4 (18.2)	18 (81.8)		6 (27.3)	16 (72.7)		0	22 (100.0)	
Mastectomy	13 (59.1)	9 (40.9)		8 (36.4)	14 (63.6)		8 (36.4)	14 (63.6)		0	22 (100.0)	
Lymph node excision	11 (73.3)	4 (26.7)		2 (13.3)	13 (86.7)		3 (20.0)	12 (80.0)		0	15 (100.0)	
Vascular surgeries	4 (8.9)	41 (91.1)		11 (24.4)	34 (75.6)		17 (37.8)	28 (62.2)		0	45 (100.0)	
Duration of operation												
≤ 30 min	33 (49.3)	34 (50.7)		21 (31.3)	46 (68.7)		18 (26.9)	49 (73.1)		0	67 (100.0)	
31 to 60 min	61 (46.9)	69 (53.1)		61 (46.9)	69 (53.1)		55 (42.3)	75 (57.7)		4 (3.1)	126 (96.9)	
61 to 90 min	32 (82.1)	7 (17.9)	**0.001**	12 (30.8)	27 (69.2)	0.17	14 (35.9)	25 (64.1)	0.28	0	39 (100.0)	0.50
91 to 120 min	28 (68.3)	13 (31.7)		16 (39.0)	25 (61.0)		17 (41.5)	24 (58.5)		1 (2.4)	40 (97.6)	
> 120 min	2 (50.0)	2 (50.0)		1 (25.0)	3 (75.0)		2 (50.0)	2 (50.0)		0	4 (100.0)	
Urgency												
Elective	131 (63.0)	77 (37.0)	**< 0.001**	60 (28.8)	148 (71.2)	**< 0.001**	65 (31.2)	143 (68.8)	**< 0.001**	1 (0.5)	207 (99.5)	**0.017**
Emergency	25 (34.2)	48 (65.8)		51 (69.9)	22 (30.1)		41 (56.2)	32 (43.8)		4 (5.5)	69 (94.5)	
Inraoperative contamination												
Clean	70 (48.6)	74 (51.4)		43 (29.9)	101 (70.1)		48 (33.3)	96 (66.7)		1 (0.7)	143 (99.3)	
Clean-contaminated	57 (67.9)	27 (32.1)	**0.002**	34 (40.5)	50 (59.5)	**< 0.001**	29 (34.5)	55 (65.5)	**0.013**	3 (3.6)	81 (96.4)	0.39
Contaminated	24 (66.7)	12 (33.3)		21 (58.3)	15 (41.7)		17 (47.2)	19 (52.8)		1 (2.8)	35 (97.2)	
Dirty	5 (29.4)	12 (70.6)		13 (76.5)	4 (23.5)		12 (70.6)	5 (29.4)		0	17 (100.0)	
Hospital												
X	62 (55.9)	49 (44.1)	**< 0.001**	19 (17.1)	92 (82.9)	**< 0.001**	27 (24.3)	84 (75.7)	**< 0.001**	5 (4.5)	106 (95.5)	**0.020**
Y	33 (30.3)	76 (69.7)		83 (76.1)	26 (23.9)		79 (72.5)	30 (27.5)		0	109 (100.0)	
Z	61 (100.0)	0		9 (14.8)	52 (85.2)		0	61 (100.0)		0	61 (100.0)	
Time of operation												
December 2016	16 (19.3)	67 (80.7)	**< 0.001**	56 (67.5)	27 (32.5)	**< 0.001**	43 (51.8)	40 (48.2)	**< 0.001**	4 (4.8)	79 (95.2)	**0.040**
January 2017	54 (62.1)	33 (37.9)		32 (36.8)	55 (63.2)		36 (41.4)	51 (58.6)		0	87 (100.0)	
February 2017	86 (77.5)	25 (22.5)		23 (20.7)	88 (79.3)		27 (24.3)	84 (75.7)		1 (0.9)	110 (99.1)	

*n* number of patients tested, *ASA* American Society of Anaesthesiologists, *FiO*_*2*_ fraction of inspired oxygen

Note: *p*-values in bold are statistically significant.

## Discussion

Procedure-related factors, such as type of operation and urgency of the procedure, displayed stronger associations with adherence to SSI preventive measures than patient-related factors, such as gender, ASA classification or age, which demonstrated little or no association with adherence to SSI preventive measures. Furthermore, location (hospital settings) and time of the operation had significant associations with adherence to all preventive measures except for surgical antibiotic prophylaxis. Moreover, the type of operation had a significant association with performing all measures except changing surgical instruments. On the other hand, patient age, duration of operation and intraoperative contamination had surprisingly little or no association with adherence to SSI preventive measures, despite possibly influencing SSI risk. Interestingly, surgical antibiotic prophylaxis was given in a very high proportion of operations (96.7%), regardless of indication. Therefore, clinical guidelines and knowledge of SSI risk factors could make surgeons a major factor in better adherence to SSI preventive measures and, thus, leading to better outcomes of surgical procedures by reducing the postoperative complication of SSI.

### Patient age and application of SSI preventive measures

Studies that examined SSI risk factors have shown that most SSIs are attributable to patient-related factors rather than procedure-related factors [20, 21]. Age is one of these major factors. Advanced age is considered as a significant risk factor for SSI [5]. However, controversy around the impact of age on the risk of SSI persists with some studies suggesting no association of advanced age with occurrence of SSI [17, 22–24]. The lack of significant associations between the patient age-group and the surgeon’s adherence to any measure, could be due to an awareness of surgeons of this controversy about the potential of age as a risk factor for SSI influencing their decision and causing their practice to deviate from the guidelines. However, even if age group does not have a direct association, it can interplay with other factors related to the procedure. Older patients tend to have more frequent emergency surgeries than younger ones and longer operating times [16]. This study shows that both of these factors increase the likelihood of poor adherence to SSI prevention guidelines, with surgeons demonstrating poorer adherence in emergency surgeries and with longer operating times. This is paradoxical, as in these situations greater adherence could possibly mitigate the greater risk. Fatigue with longer operating times as well as less support staff in emergencies could be reasons for this paradox and one avenue to reduce SSI risk could be a reduction of the impact of fatigue and poorer support on physician behaviour.

### Location and time of the surgical procedure

The risk of SSI is higher in LMICs compared to high-income countries [25] and this varies widely between procedures, surgeons, patients and hospitals [21]. In an international study, patients in LMICs were more likely to receive surgical antibiotic prophylaxis than those in high-income countries [3]. This could be explained by the fact that surgeons are aware about the higher rate of SSI in LMICs. However, this can increase the financial burden and the development of bacterial resistance or even SSI [26]. Furthermore, LMICs might lack expertise and resources required for effective surveillance of SSI which may lead to underestimation of their occurrence, resulting in a higher incidence than those reported [9, 27].

In this study, the hospital and timing in which the procedure was performed had a significant association with adherence to every tested SSI preventive measure, suggesting that shared work culture has a large impact on adherence rates. Similar findings were observed in adherence rates to obstetric guidelines in a different study [28]. This opens a door to using the effectiveness of facility-based interventions in order to improve adherence to SSI prevention measures. The effectiveness of interventions varies greatly in different settings and is highly dependent on the context [29–31]. Therefore, facility-based improvement strategies can be effective and be adjusted to dynamic contexts through continuous evaluation and, thus, pose an effective tool for healthcare quality improvements also in low-resource settings [32]. However, it remains unclear why the adherence to SSI preventive measures generally improved over the months of the study. Surgeons and data collectors were blinded to the purpose of the observations in order to reduce potential bias from the Hawthorne effect, when clinicians ‘improve’ their practice, because they are conscious of being observed [14, 15]. However, it cannot be excluded, that surgeons ‘learned’ to improve adherence to SSI preventive measures over time.

### Surgical antibiotic prophylaxis

Surgical antibiotic prophylaxis is one of the most effective methods to prevent SSIs [26]. However, a rationale should exist while prescribing these antibiotics to avoid bacterial resistance and high treatment costs. The WHO guidelines [8] aim to achieve this, but several studies have shown that adherence is not optimal [33–35]. In comparison to a Brazilian study [15], this study found a higher rate of administering surgical antibiotic prophylaxis (96.0% vs 77.6%), which was comparable to an Italian study [16] (96.0% vs 96.8%). The effectivity of surgical antibiotic prophylaxis may depend on the type of surgery performed [36], and as a result, if a surgeon is aware of this, the adherence to SSI prevention guidelines might be expected to be improved. The Sanford Guide on Antimicrobial Therapy recommends antibiotic prophylaxis in all gastrointestinal surgeries, mastectomy with lymph node dissection and vascular surgery of upper and lower limb [37]. Therefore, in this study, antibiotic prophylaxis appears to have been overused in up to 42 cases (14.9%), as in thyroidectomy and mastectomy, while it was not given in nine surgeries (3.2%), which might have benefitted from receiving prophylaxis. One factor for the potentially inappropriate use of prophylactic antibiotics in 18% of surgeries might be the lack of clear local and national guidelines for antibiotic prophylaxis. Furthermore, this indicates lack of awareness among surgeons regarding antibiotic prophylaxis or a lack of appropriate observance of SSI preventive measures. Similar findings of overuse and underuse of antibiotic prophylaxis were also reported by other studies [34, 38, 39]. However, in this study more than 80% of surgeries were performed with appropriate antibiotic prophylaxis, although overuse was possibly also expressed in the fact that 30 patients (10.7%) received antibiotics twice; within 120 min before surgery as well as again at the time of incision. Previous studies have shown inappropriate adherence to infection control measures on local neonatal intensive care units, which could be improved after introduction of national guidelines and staff training measures, suggesting deficiencies in awareness of guidelines among local surgeons being a significant factor in poor adherence to SSI preventive measures [40].

Further factors influencing the use of prophylactic antibiotics could be the duration of the operation and the usage of drains [41, 42]. The impact of the duration of surgery on SSI risks as well as the effect of prophylactic antibiotics on reducing such risks remains controversial [42, 43]. Longer operating times may lead to surgeon fatigue, which might compound non-observance of SSI preventive measures [44]. Prophylactic antibiotics were also shown to be less effective in laparoscopic than open cholecystectomy [45], which might influence the decision on using antibiotic prophylaxis. However, in this sample, 58 of 59 laparoscopic cholecystectomies received antibiotics, confirming the strong culture among local surgeons on using prophylactic antibiotics, as reflected in the high percentage of prophylactic antibiotics given across hospitals and surgeries.

### Change of or double gloves

In contrast to the current study, double gloving was significantly associated with longer duration of operation in a study in Brazil, possibly due to the greater risk of glove perforation with longer duration of operations [15]. It is expected that the need for change of or double gloving in this study is underestimated. This is due to the probability that surgeons did not realize that they had their gloves perforated, causing many to use them until the end of surgery without changing gloves or seeing the need for double gloving [46]. Moreover, the lack of evidence to support the change of or double gloves might have convinced surgeons against the use of two gloves. Agreed local practice guidelines might help clinicians in this situation to make the correct choices, also demonstrating the impact that greater awareness of SSI preventive measures could have on reducing SSI rates.

### Hair removal

Compared to a study in Brazil [47], hair removal was noted to be done slightly more frequently in this study (20.0% vs 26.7%). According to international guidelines [8], patients should not have their hair removed unless the presence of the hair interferes with the surgery. If necessary, the use of electric clippers to remove hair from the incision site is recommended immediately prior to surgery. However, in this study, hair removal was done preoperatively by shaving or even, in three cases, surgeons used the surgical knife for hair removal from the operating site. Shaving and hair removal in the operating room are clearly not recommended and represent non-adherence to the guidelines for SSI preventive measures. It is not clear if these findings are due to electric clippers not being available or to a lack of knowledge of guidelines among surgeons or a combination of both. On the other hand, hair removal was only performed in around one quarter of patients, mostly men, and a large proportion of open hernia repairs (60.5%), open appendectomies (45.7%) and vascular surgeries (33.3%), indicating that these included cases necessitating hair removal due to hairiness, which is more common in men than women and the stated surgical procedures possibly affect hairy areas on the lower abdomen and limbs. This suggests, at least, partial awareness of guidelines and use of shaving due to lack of other options.

### Use of anti-septic agents

The choice of agent for skin preparation or hand washing often depends on which agents are available at the time, as availability of such equipment fluctuates in Gaza and is not constant. Therefore, lack of evidence in this matter, could also be a reason for the Palestinian Ministry of Health not making adequate materials available for these measures, but to prioritise usage of the very limited funds and resources for practices supported by actual evidence. However, clear guidance for clinicians will facilitate common practice and observance of guidelines, which, combined with staff training, could lead to improved adherence to SSI preventive measures and sustained reduction of SSIs [40, 47–49].

The major strength of this study lies in its attempt to find a novel approach to the problem of SSI and improvement of adherence to prevention guidelines. Interestingly, a previous study [50] showed that even if an optimal adherence to the current surgical care improvement project measures was achieved, this would only result in a < 25% decrease in the SSI rates. Therefore, it is important to find new ways to approach this important subject.

Limitations include the lack of postoperative follow-up to evaluate the association between non-adherence to infection prevention measures and the occurrence of SSI. Furthermore, the number of operations included in the study was small preventing sophisticated data analysis (e.g, by multivariate logistic regression) that can be used to adjust for patient-related and procedure-related factors as well as to test for interactions between them. In addition, a formal sample size calculation had not been done due to the study design being descriptive-observational and focusing on healthcare staff behaviours. The observational nature of this study further limited expansion of the sample. Moreover, this study did not address all the recommendations for the prevention and control of SSIs, such as cleaning and disinfection of environmental surfaces, sterilization of surgical instruments and surgeon-related factors. However, it examined the most important aspects of local practice that need more attention and improvement by creating an infection-control policy that can be followed especially in resource-limited settings as in the Gaza Strip. Last but not least, there is a lack of reporting of sociodemographic data, economic and health status, which can influence the patients’ adherence to some preventive measures like antibiotic prescription [51].

## Conclusion

This study shows that surgeons might be a major factor in improving adherence rates to SSI preventive measures. Therefore, improving surgeons’ awareness and knowledge by regular training and education sessions highlighting the importance of SSI preventive measures in clinical practice might improve the quality of care provided to surgical patients [47].

There is an urgent need for the development of local guidelines as well as promotion of awareness and knowledge of evidence-based practice among surgical staff. Conducting regular audits and giving feedback to the staff regarding the implementation of policy recommendations can contribute to increasing adherence. Workplace culture has a significant impact on adherence to SSI preventive measures, which underlines the key role of hospital leadership as well as surgical teams in reducing SSI. Further research is needed to more rigorously evaluate the effectiveness of new strategies to increase adherence to SSI preventive measures and their impact on actual SSI rates.

## Data Availability

Data can be made available under reasonable request from the corresponding author.

## Abbreviations

ASA: American Society of Anaesthesiologists
FiO_2_: Fraction of inspired oxygen
LMICs: Low- and middle-income countries
SPSS: Statistical Package for the Social Sciences
SSI: Surgical site infection
WHO: World Health Organization

## References

[1] Centers for Disease Control and Prevention. Surgical Site Infection (SSI) Event. Available from:https://www.cdc.gov/nhsn/pdfs/pscmanual/9pscssicurrent.pdf. Accessed 22 May 2019.

[2] Allegranzi B, Bagheri Nejad S, Combescure C, Graafmans W, Attar H, Donaldson L (2011). Burden of endemic health-care-associated infection in developing countries: systematic review and meta-analysis. Lancet..

[3] GlobalSurg Collaborative (2018). Surgical site infection after gastrointestinal surgery in high-income, middle-income, and low-income countries: a prospective, international, multicentre cohort study. Lancet Infect Dis.

[4] Alser O, Tahboub H, Al-Slaibi I, Abuowda Y, Elshami M, Albarqouni L (2019). Surgical site infections following gastrointestinal surgery in Palestine: a multicentre, prospective cohort study. Lancet.

[5] Korol E, Johnston K, Waser N, Sifakis F, Jafri HS, Lo M (2013). A systematic review of risk factors associated with surgical site infections among surgical patients. PLoS One.

[6] Hubner M, Diana M, Zanetti G, Eisenring MC, Demartines N, Troillet N (2011). Surgical site infections in colon surgery: the patient, the procedure, the hospital, and the surgeon. Arch Surg.

[7] Carvalho RLR, Campos CC, Franco LMC, Rocha AM, Ercole FF (2017). Incidence and risk factors for surgical site infection in general surgeries. Rev Lat Am Enfermagem.

[8] Allegranzi B, Zayed B, Bischoff P, Kubilay NZ, de Jonge S, de Vries F (2016). New WHO recommendations on intraoperative and postoperative measures for surgical site infection prevention: an evidence-based global perspective. Lancet Infect Dis.

[9] Aiken AM, Karuri DM, Wanyoro AK, Macleod J (2012). Interventional studies for preventing surgical site infections in sub-Saharan Africa - a systematic review. Int J Surg.

[10] Allegranzi B, Pittet D (2007). Healthcare-associated infection in developing countries: simple solutions to meet complex challenges. Infect Control Hosp Epidemiol.

[11] Shears P (2007). Poverty and infection in the developing world: healthcare-related infections and infection control in the tropics. J Hosp Infect.

[12] Durando P, Bassetti M, Orengo G, Crimi P, Battistini A, Bellina D (2012). Adherence to international and national recommendations for the prevention of surgical site infections in Italy: results from an observational prospective study in elective surgery. Am J Infect Control.

[13] Palestinian Ministry of Health. Annual Report for Healthcare in the Gaza Strip 2017. Available from: http://www.moh.gov.ps/portal/wp-42 content/uploads/2018/08/MOH-Annual-Report-2017-Final-9-9-2018. Accessed 22 May 2019.

[14] Gould DJ, Drey NS, Creedon S (2011). Routine hand hygiene audit by direct observation: has nemesis arrived?. J Hosp Infect.

[15] Oliveira AC, Gama CS (2015). Evaluation of adherence to measures for the prevention of surgical site infections by the surgical team. Rev Esc Enferm USP.

[16] Agodi A, Quattrocchi A, Barchitta M, Adornetto V, Cocuzza A, Latino R (2015). Risk of surgical site infection in older patients in a cohort survey: targets for quality improvement in antibiotic prophylaxis. Int Surg.

[17] Kaye KS, Schmit K, Pieper C, Sloane R, Caughlan KF, Sexton DJ (2005). The effect of increasing age on the risk of surgical site infection. J Infect Dis.

[18] Cheng H, Chen BP, Soleas IM, Ferko NC, Cameron CG, Hinoul P (2017). Prolonged operative duration increases risk of surgical site infections: a systematic review. Surg Infect.

[19] Fan Y, Wei Z, Wang W, Tan L, Jiang H, Tian L (2014). The incidence and distribution of surgical site infection in mainland China: a meta-analysis of 84 prospective observational studies. Sci Rep.

[20] Owens CD, Stoessel K (2008). Surgical site infections: epidemiology, microbiology and prevention. J Hosp Infect.

[21] Poggio JL (2013). Perioperative strategies to prevent surgical-site infection. Clin Colon Rectal Surg.

[22] Haley RW, Hooton TM, Culver DH, Stanley RC, Emori TG, Hardison CD (1981). Nosocomial infections in U.S. hospitals, 1975-1976: estimated frequency by selected characteristics of patients. Am J Med.

[23] Olsen MA, Lock-Buckley P, Hopkins D, Polish LB, Sundt TM, Fraser VJ (2002). The risk factors for deep and superficial chest surgical-site infections after coronary artery bypass graft surgery are different. J Thorac Cardiovasc Surg.

[24] Pessaux P, Msika S, Atalla D, Hay JM, Flamant Y, French Association for Surgical R (2003). Risk factors for postoperative infectious complications in noncolorectal abdominal surgery: a multivariate analysis based on a prospective multicenter study of 4718 patients. Arch Surg.

[25] Rosenthal VD, Maki DG, Graves N (2008). The international nosocomial infection control consortium (INICC): goals and objectives, description of surveillance methods, and operational activities. Am J Infect Control.

[26] Mehta JA, Sable SA, Nagral S (2015). Updated recommendations for control of surgical site infections. Ann Surg.

[27] Bagheri Nejad S, Allegranzi B, Syed SB, Ellis B, Pittet D (2011). Health-care-associated infection in Africa: a systematic review. Bull World Health Organ.

[28] Utsumi M, Shimizu J, Miyamoto A, Umeshita K, Kobayashi T, Monden M (2010). Age as an independent risk factor for surgical site infections in a large gastrointestinal surgery cohort in Japan. J Hosp Infect.

[29] Coles E, Wells M, Maxwell M, Harris FM, Anderson J, Gray NM (2017). The influence of contextual factors on healthcare quality improvement initiatives: what works, for whom and in what setting? Protocol for a realist review. Syst Rev.

[30] Kaplan HC, Brady PW, Dritz MC, Hooper DK, Linam WM, Froehle CM (2010). The influence of context on quality improvement success in health care: a systematic review of the literature. Milbank Q.

[31] Walshe K, Freeman T (2002). Effectiveness of quality improvement: learning from evaluations. Qual Saf Health Care.

[32] Pirkle CM, Dumont A, Zunzunegui MV (2011). Criterion-based clinical audit to assess quality of obstetrical care in low- and middle-income countries: a systematic review. Int J Qual Health Care.

[33] Barchitta M, Matranga D, Quattrocchi A, Bellocchi P, Ruffino M, Basile G (2012). Prevalence of surgical site infections before and after the implementation of a multimodal infection control programme. J Antimicrob Chemother.

[34] Friedman ND, Styles K, Gray AM, Low J, Athan E (2013). Compliance with surgical antibiotic prophylaxis at an Australian teaching hospital. Am J Infect Control.

[35] Tourmousoglou CE, Yiannakopoulou E, Kalapothaki V, Bramis J, St Papadopoulos J (2008). Adherence to guidelines for antibiotic prophylaxis in general surgery: a critical appraisal. J Antimicrob Chemother.

[36] Liu Z, Dumville JC, Norman G, Westby MJ, Blazeby J, McFarlane E (2018). Intraoperative interventions for preventing surgical site infection: an overview of Cochrane reviews. Cochrane Database Syst Rev.

[37] Gilbert DN, Chambers AF, Eliopoulos GM, Saag MS, Black D, Freedman DO, Kim K, Schwartz BS (2018). The Sanford guide to antimicrobial therapy 2018.

[38] Magill SS, Edwards JR, Bamberg W, Beldavs ZG, Dumyati G, Kainer MA (2014). Multistate point-prevalence survey of health care-associated infections. N Engl J Med.

[39] Poeran J, Wasserman I, Zubizarreta N, Mazumdar M (2016). Characteristics of antibiotic prophylaxis and risk of surgical site infections in open colectomies. Dis Colon Rectum.

[40] Alrumi N, Aghaalkurdi M, Habib H, Abed S, Bottcher B. Infection control measures in neonatal units: implementation of change in the Gaza-strip. J Matern Fetal Neonatal Med. 2019:1–7. 10.1080/14767058.2019.1576168. 10.1080/14767058.2019.1576168 30691321

[41] Degnim AC, Scow JS, Hoskin TL, Miller JP, Loprinzi M, Boughey JC (2013). Randomized controlled trial to reduce bacterial colonization of surgical drains after breast and axillary operations. Ann Surg.

[42] Leong G, Wilson J, Charlett A (2006). Duration of operation as a risk factor for surgical site infection: comparison of English and US data. J Hosp Infect.

[43] Dornfeld M, Lovely JK, Huebner M, Larson DW (2017). Surgical site infection in colorectal surgery: a study in antibiotic duration. Dis Colon Rectum.

[44] Li GQ, Guo FF, Ou Y, Dong GW, Zhou W (2013). Epidemiology and outcomes of surgical site infections following orthopedic surgery. Am J Infect Control.

[45] Bratzler DW, Houck PM, Surgical Infection Prevention Guideline Writers W (2005). Antimicrobial prophylaxis for surgery: an advisory statement from the National Surgical Infection Prevention Project. Am J Surg.

[46] Korniewicz D, El-Masri M (2012). Exploring the benefits of double gloving during surgery. AORN J.

[47] de Oliveira AC, Sarmento GC (2017). Surgical site infection prevention: an analysis of compliance with good practice in a teaching hospital. J Infect Prev.

[48] Elshami M, Dabbour R, Alkhatib M, Abdalghafoor T, Alaloul E, Habib M (2019). Evaluating the adherence to guidelines for management of acute heart failure in the Gaza strip hospitals: a medical chart–based review study. Glob J Qual Saf Healthc.

[49] Zimmo M, Laine K, Hassan S, Fosse E, Lieng M, Ali-Masri H (2018). Differences in rates and odds for emergency caesarean section in six Palestinian hospitals: a population-based birth cohort study. BMJ Open.

[50] Stulberg JJ, Delaney CP, Neuhauser DV, Aron DC, Fu P, Koroukian SM (2010). Adherence to surgical care improvement project measures and the association with postoperative infections. JAMA..

[51] Osterberg L, Blaschke T (2005). Adherence to medication. N Engl J Med.

